# “Save for a rainy day” or “live in the moment”? How does uncertainty associated with earthquakes affect people’s time preferences?

**DOI:** 10.3389/fpsyg.2022.1039092

**Published:** 2022-11-11

**Authors:** Congming Ding, Xueying Yan, Zhiyuan Chen

**Affiliations:** School of Public Policy and Administration, Chongqing University, Chongqing, China

**Keywords:** earthquake frequency, uncertainty, time preferences, savings rate, education investment, consumption type, mindset

## Abstract

Uncertainty caused by frequent earthquakes can permanently reshape people’s time preferences, forcing them to confront the question of whether to “save for a rainy day” or “live in the moment.” Focusing on China, this study empirically analyzes the effect of earthquake frequency on local residents’ time preferences, using seismic data from 780 BCE to 1970 CE matched to the China Family Panel Studies (2010) database (CFPS). The results show that uncertainty arising from earthquakes continuously influences residents’ time preferences and behavior. Specifically, in regions with a higher earthquake frequency, residents’ saving rate is lower and households’ consumption level is higher, suggesting that people exposed to frequent earthquakes pay more attention to the present than the future. The results further show that residents’ education investment level and average education level are lower in higher earthquake frequency regions. The empirical evidence demonstrates that differences in mindset are the primary reason for the observed regional differences in consumption, savings and education. The results of a series of robustness tests demonstrate the robustness of the above-listed findings. This research sheds new light on the relationship between natural disasters and human time preferences.

## Introduction

The development of human society is closely related to natural disasters. Earthquakes are one of the most common types of natural disasters and thus exert a significant economic, political, and cultural impact. This impact is evident both at the macro level, as evidenced by post-disaster reconstruction, economic loss estimation and disaster relief, and at the micro level, characterized by changes in time preferences and psychological trauma (Kun et al., 2013; Callen, 2015; Filipski et al., 2015; Yao et al., 2019a,b). Uncertainty due to earthquakes can have immediate and devastating effects on a region and a country, as well as profound and lasting effects that are transmitted from generation to generation (Zhang and Liu, 2021; Yu, 2022). Uncertainty refers to the situation that socio-economic entities cannot guarantee the distribution range and status of future economic conditions (Knight, 1921).

Time preference refers to the fact that for the same consumption bundle, people always prefer the present to the future. Time preference is widely involved in consumption, saving and investment. The time preference rate is calculated as the marginal rate of substitution between current and future consumption (Li et al., 2012). The savings rate in Chinese society tends to be high due to the historical influence of a traditional small-holder economy, which is characterized by an emphasis on self-sufficiency and Confucian cultural traditions. In terms of time preferences, Chinese people generally prefer future consumption to current consumption. In comparison, European and American societies generally have higher consumption and lower savings rates, and members of these societies tend to prioritize present over future consumption. Ye et al. (2012) find that over-consumption is prevalent in Europe and the United States, whereas under-consumption is prevalent in East Asia. Clearly, factors such as the consumption culture and time preferences influence these observed systematic consumption preferences.

In this paper, we explore the impact of frequent historical earthquakes on people’s current time preferences. Specifically, we ask whether frequent historical earthquakes change people’s time preferences and alter their consumption and savings behavior. Our findings confirm that people in regions with frequent earthquakes tend to adopt a “living for today” mentality, which drives an increase in consumption and reductions in savings and investment in personal human capital. The main innovations of this paper are as follows. First, it enriches the study of microeconomic uncertainty associated with earthquakes from a historical perspective, in contrast with earlier studies on disasters that focus mainly on macroeconomic aspects, such as related losses and the demands of affected residents (Kelly, 2008). In recent years, however, the enrichment of micro-level data has led to an increase in studies of earthquakes and microeconomic behavior, such as the impact of earthquakes on various aspects of time and risk preferences (Yao et al., 2019a; Zhang and Liu, 2021; Yu, 2022; Zhang et al., 2022). Many scholars study the factors influencing savings, such as new social security system, fiscal spending, income, and reference point dependence. In contrast with these studies, we study the relationship between uncertainty due to earthquakes and saving and consumption behavior from a longer historical perspective. Second, this paper adds to the literature on earthquakes and time preferences through a new approach. Currently, research on natural disasters and time preferences tends to explore the impact of a single natural disaster on time preferences (Becchetti et al., 2012; Cameron and Shah, 2012; Callen, 2015; Cassar et al., 2017) or to use exposure to earthquakes during a short period of time to study the impact of earthquake experiences on savings (Zhang and Liu, 2021). These studies of the impacts of natural hazards are rigorous and have good explanatory power. However, some areas in China are located in seismic zones and their residents have been exposed to earthquakes for a long time; that is, the effects of earthquakes on these areas are historical and long-term. If we were to study time preferences in the context of a single earthquake or short-term earthquake exposure, we would ignore the long-term historical effects of earthquakes and risk overestimating the effects of a single event.

Zhang and Liu (2021) study the long-term effects of earthquakes on urban residents’ savings and consumption habits. They conclude that their “living in the present” hypothesis better characterizes the long-term effects of earthquake experience on household savings and consumption habits than their “saving for a rainy day” hypothesis. Although Zhang and Liu’s findings are similar to our findings in this paper, we highlight some differences between the studies. First, Zhang and Liu use data on earthquakes with a magnitude of 4.5 or greater that occurred in 18 sampled provinces and cities from 1900 to 2009. In contrast, we use data from a longer period (780 BCE to 1970 CE) to better portray the profound historical, cultural, and conceptual effects of earthquake uncertainty on a region. Second, Zhang and Liu use the frequency of earthquakes with a magnitude of 4.5 or greater experienced by household heads as the core explanatory variable, with memory as their main measure of the degree of earthquake shocks suffered by urban households and individuals. In contrast, we sum the historical number of earthquakes in each district and county to measure the extent to which the region has been affected by earthquakes. We also test changes in time preferences throughout a region; specifically, we test the long-term historical impact of earthquakes on the development of time preferences across the region. Finally, whereas Zhang and Liu mainly explore changes in savings and consumption, we also evaluate the effects of historical earthquakes on human capital investment, which also reflect changes in time preferences.

In this paper, we construct historical earthquake indicators using cataloged Chinese historical earthquake data and time preference-related indicators from the CFPS (2010) database. Our empirical analysis yields several results. (1) Historically, earthquakes have negative and positive effects on time preferences. Specifically, they have negative effects on savings rates, education and mindset and a positive effect on consumption rates. In other words, households in regions with more earthquakes tend to have lower savings rates, higher consumption rates, fewer years of education per capita and less human capital investment than households in other regions. These differences may occur in response to influences that affect mindset. (2) Using robustness tests in which we replace the explanatory and explained variables in the model and adjust the lengths of earthquake samples, we reveal that the main findings of our study still hold. (3) Using a heterogeneity test to explore earthquake frequency and magnitude, we find that a high earthquake frequency has a more significant effect on people’s time preferences than a large seismic magnitude does. Importantly, we also find that the significant effect of earthquakes on the mindset of earthquake zone residents may explain how earthquakes affect peoples’ saving and consumption behavior.

The rest of this paper is organized as follows. In the second section, we discuss relevant facts and our theoretical hypotheses. In the third section, we present the research data and variable descriptions. In the fourth section, we present the results of our empirical analyses. In the fifth section, we present the results of robustness tests. Finally, the sixth section concludes the article.

## Relevant facts and theoretical hypotheses

### Relevant facts

Seismic activity occurring in Asia accounts for 70 to 80% of seismic activity worldwide (Zhao and Zhang, 1999). Asia has long been a hotbed of seismic activity. China is located between and influenced by the Asian–European plate and the seismic zone of the Pacific Rim plate, resulting in frequent seismic activity. Statistics show that about 35% of the world’s continental earthquakes of magnitude 7 or higher occurred in China; of the 1.2 million people who died in the 20th century due to earthquakes worldwide, China accounted for 590,000. In addition, more than 1/3 of China, nearly 1/2 of our cities and nearly 2/3 of our megacities with more than one million people are located in high seismic intensity zones above VII degrees, while the high intensity zones above VII degrees in the United States account for only 12% of its land area (Huang, 2009). Many large-scale earthquakes have occurred in China in recent decades, including the Tangshan earthquake in 1976, the Wenchuan earthquake in 2008, the Yushu earthquake in 2010, the Ya’an earthquake in 2013, and the Yingjiang earthquake in 2014. These events have caused enormous numbers of casualties and damage to homes and other property. Most earthquakes occur suddenly and are difficult to predict, especially as earthquake prediction technology is not yet perfect. Compared with other natural disasters, which may progress over weeks, earthquakes often occur within minutes or even seconds. Despite their short duration, the damage caused by an earthquake is difficult to predict. An earthquake can destroy an entire community in just a few minutes. At the same time, strong earthquakes not only destroy and collapse buildings, causing a large number of casualties and economic losses, but also often trigger a series of secondary disasters such as fires, floods, toxic gas leakage and so on. Sometimes the losses from secondary disasters of earthquakes even exceed those from direct disasters of earthquakes (Zhao et al., 2010). This unpredictability surrounding the characteristics of the earthquake itself and the resulting property losses and casualties in a region incur profound uncertainty for the affected society.

**FIGURE 1:**
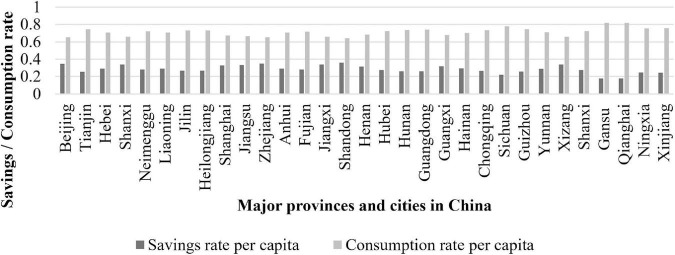
Average per capita savings rate and average per capita consumption rate of residents in major regions of China (2013–2020).

Since the reform and opening up, a focus on “high savings and low consumption” (Yin et al., 2020) has been a key factor in China’s sustained high growth and trade surplus. Although the overall savings rate is high and the overall consumption rate is low, Figure 1 shows some differences between per capita savings and consumption rates in major regions of China. Tianjin, Hebei, Sichuan, Yunnan, Tibet, Gansu, Qinghai, Ningxia, and Xinjiang have the lowest savings rates and highest consumption rates. These regions are found in the north, south, west and east of China and comprise both economically developed and developing regions. Why might regions in China have different savings and consumption rates?

**FIGURE 2:**
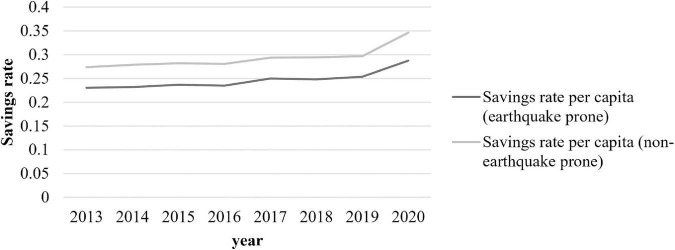
Comparison of per capita savings rate between earthquake-prone regions and non-earthquake-prone regions [The per capita savings rate is calculated using (per capita disposable income of residents−per capita consumption expenditure of residents)/per capita disposable income of residents in each province].

**FIGURE 3:**
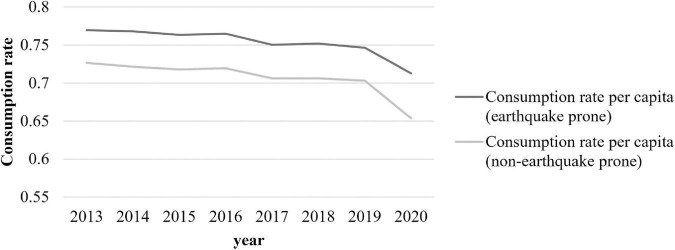
Comparison of per capita consumption rate between earthquake-prone regions and non-earthquake-prone regions (The per capita consumption rate is calculated using per capita consumption expenditure per resident/per capita disposable income of residents in each province).

Drawing on the method of Zhang and Liu (2021), we divide China into earthquake-prone and non-earthquake-prone regions to compare their average per capita consumption and savings rates.^1^ From Figures 2, 3, we can clearly see that the per capita savings rate is lower and the per capita consumption rate is higher in earthquake-prone regions than in non-earthquake prone regions.

### Current status of research

Compared to other natural hazards, earthquakes pose not only property damage but, more importantly, a more serious and immediate threat of death. While other natural hazards may pose some risk of death, the probability of death due to them is usually much smaller than economic losses. In China, deaths caused by earthquakes account for 54% of all deaths nationwide caused by all types of natural disasters (e.g., floods, hill fires, mudslides, and landslides) since the beginning of the 20th century.^2^ Earthquakes are the most serious type of natural disaster in terms of human fatalities. Other types of disaster, such as meteorological disasters, also have huge negative effects, but these effects are mainly economic. As noted previously, earthquakes are sudden, transient, and very destructive and pose a more serious and direct threat to human survival than other types of natural disasters. Data show that floods account for 40%, tropical cyclones for 20%, droughts for 15%, earthquakes for 15%, and the rest of disasters for 10% of the global losses caused by various disasters (Xu et al., 2006).

Natural disasters that cause large economic losses increase people’s risk aversion and saving behavior. Callen (2015) suggests that workers exposed to typhoon disasters are more patient than those who have not experienced typhoon disasters. As greater patience is associated with a preference for increased savings, Callen’s finding suggests that a change in time preference affects savings behavior. Cameron and Shah (2012) and Cassar et al. (2017) report that individuals who had recently been exposed to floods or earthquakes in rural Indonesia exhibited more risk aversion than those not exposed to such events. Comparing microfinance borrowers in Sri Lanka who had been affected by the 2004 tsunami with those who had not been affected, Becchetti et al. (2012) find that those who had suffered at least one associated injury (including financial losses and human casualties) behaved less altruistically as senders than those who did not report any such injury, reflecting a tendency for risk aversion among the affected borrowers. Similarly, Eckel et al. (2009) find that tsunami victims are more risk-averse than their unaffected peers.

In contrast, earthquakes that result in high mortality rates may encourage people to value the present and recognize the brevity of life. As a result, they may change their time preferences, increase their consumption in the present and reduce their savings rate. Accordingly, “living in the present” is a more appropriate description than “saving for a rainy day” to characterize the long-term effects of earthquake experiences on household savings and consumption habits (Zhang and Liu, 2021). Distinguishing between respondents with different income profiles and those who reside in rural or urban areas, Zhang and Liu (2021) find that individuals who have been severely affected by disaster and have low incomes exhibit reduced cognitive self-control, leading to an increase in current consumption and a reduction in savings. Filipski et al. (2015, 2019) empirically analyze post-earthquake changes in the savings and consumption behavior of households near the epicenter and find that these households exhibit reduced savings, increased spending on alcohol and cigarettes and an increased frequency of playing mahjong. The earthquake appears to have caused a shift in preferences characterized by households’ attitudinal preferences for just-in-time consumption in the present, and this shift may be influenced by emotional shocks and changes in risk perception.

In addition to the aforementioned studies on “valuing the present,” the literature suggests that people increase their savings after earthquakes due to prudent precautionary motives or changes in time preferences. Kun et al. (2013) demonstrate that the 2008 Wenchuan earthquake elicited severe psychological stress responses among lower-income groups, women and older people. These psychological stress responses eventually resulted in an increase in savings and decrease in consumption. Yao and Xu (2018) demonstrate an increase in precautionary saving after the 2008 Wenchuan earthquake; specifically, they find that the earthquake positively affected both short-term and longer-term saving behavior. Yao et al. (2019a) study the relationship between post-disaster experience, time preferences and household savings from a psychological perspective. They argue that self-control and cognitive ability have a combined effect on changes in people’s time preferences after an earthquake disaster, leading to an increase in savings.

The above-mentioned articles explore the short-term effects of earthquakes. In contrast, we aim to contribute a new historical perspective to the debate on the time preference of earthquakes by studying earthquakes over a long period. Durante (2009) proposes that, historically, changes in social trust levels are determined by climate change, with long-term effects. Li and Li (2020) also suggest that experiences with infectious disease can negatively affect people’s social trust levels over time; they observe the largest effect at 1–4 years after the event, followed by a diminishing effect at 5–9 years. These findings suggest that disaster experiences may have long-term impacts at the micro (i.e., individual) level.

In their studies of saving and consumption behavior, Shi and Zhu (2004), Yi et al. (2008), Du and Liu (2011), and Ma and Zhou (2014) identify some precautionary motives for saving in China and observe that these motives are stronger in western rural areas than in central and eastern regions. Giles and Yoo (2007) and Lu et al. (2014) both find that non-farm employment and human capital in rural households can reduce precautionary savings by reducing the shocks from earthquake disasters. In contrast with these papers, which illustrate the existence of precautionary savings in China, Filipski et al. (2019) find that earthquakes elicit a stronger propensity to “live for today” than to engage in precautionary saving. This means that although people save as a precaution, they are likely to save less and increase their consumption when exposed to prolonged turbulence. In terms of research on human capital investment behavior, both Paudel and Ryu (2018) and Tian et al. (2022) find that earthquakes have a negative impact on people’s human capital investment.

Drawing on the above analyses, we propose the following hypothesis. The impact of historical earthquakes on people’s time preferences is influenced by the “living for today” mentality, leading to an increased concern for the present. The psychological impact of this mentality is demonstrated by lower savings rates, higher consumption rates and less human capital investment among households in regions with more earthquakes than among households in regions with fewer earthquakes.

## Description of data and variables

### Data sources

The primary data used in this study are obtained from the CFPS (2010) database, which includes basic survey data from 14,789 Chinese households located in 635 villages in 162 counties across 25 provinces. The database comprises more than 10,000 household data samples and includes detailed information about household incomes, total household expenditures, genealogical ancestral halls, values and social status (Ding et al., 2018). The multi-stage stratified sampling design of the CFPS enables representation of approximately 95% of the Chinese population (Xie, 2012). This rich dataset provides a good basis for our study. We also use historical earthquake catalog data pertaining to China from the National Center for Earthquake Science and Data, which records data on earthquakes, including time, latitude, longitude, depth, magnitude and intensity, from 780 BCE to 1979 CE. This dataset contains 6,053 events. To meet the needs of our empirical study, we mainly select household data and adult data from CFPS and match these with the historical earthquake data, yielding 2,456 successfully matched data samples. Data samples with missing total household expenditure, income and household retirement income values in the household questionnaire panel are removed.

### Core variables and sample selection^3^

This paper focuses on the effect of historical earthquakes on people’s time preferences. We conjecture that sustained exposure to earthquakes elicits a mindset of “living for today” and leads people to prefer hedonistic consumption and to reduce their savings and investment in their own development. To support this conjecture, we select three areas for further study: savings, consumption and education. We also use mindset area for further test. Table 1 shows the descriptive statistics of all variables.

**TABLE 1:** Descriptive statistics of each variable.

Variables	Obs	Mean	Std. Dev.	Min	Max
**Key explanatory variables**					
Earthquakes frequency	2456	3.342	3.133	1.000	12.000
Recent earthquake time	2456	7.387	0.430	5.841	7.590
Interaction item: magnitude * time	2398	9.056	0.519	7.545	9.548
Interaction item: frequency * magnitude	2456	2.504	0.851	1.386	4.199
**Explained variable-savings**					
Saving	2164	0.001	0.932	−3.667	0.838
Sav-edu&med	2164	−0.29	1.267	−5.350	0.790
**Explained variable-consumption**					
Consumption	2164	0.999	0.932	0.162	4.667
Cons-edu&med	2164	1.334	1.267	0.210	6.350
Cons-spending	2188	1.120	1.044	0.169	5.376
Cons-survival	2262	0.505	0.444	0.061	2.250
Cons-enjoyment	2331	0.243	0.759	0.000	4.000
Cons-development	2289	0.236	0.285	0.000	1.256
**Explained variable-education**					
Education	2456	8.324	1.410	4.830	11.080
Education expenditure	2431	0.117	0.210	0.000	2.941
**Explained variable-ideology**					
Religious-day	130	1.309	1.522	0.100	12.000
Religious-end	141	1.433	1.448	0.100	10.000
Fitness	816	1.472	0.789	1.000	5.000
Opinion: Intelligence	6599	3.192	0.944	1.000	6.000
Importance: Fun	6599	3.974	0.980	1.000	5.000
Importance: Offspring	6599	4.088	1.090	1.000	5.000
Importance: Children	6599	4.540	0.806	1.000	5.000
**Characteristics variables: household**					
Family income	2331	30.343	44.787	0.005	1406.000
Family size	2456	4.419	1.763	1.000	15.000
Health	2435	3.399	8.699	0.000	155.000
**Characteristics variables: region**					
Population	2456	5.461	4.126	0.555	23.365
non-agricultural population	2456	21.612	18.760	4.160	82.140
**Characteristics variables: economic**					
GDP	2456	9.386	0.880	8.324	10.737
GDP per person	2456	10.129	0.457	9.665	11.198
Cincome	2456	30.413	15.505	8.435	107.928

Sources: (1) village average income for the explanatory variables, household characteristics variables, regional characteristics variables, and economic characteristics variables were obtained from the CFPS database, all selected from 2010 data; (2) data for the explanatory variables were obtained from the National Seismological Science Data Center’s historical earthquake catalog of China; (3) GDP and GDP per capita for the economic characteristics variables at the provincial level were obtained from the EPS data platform.

#### Measurement of savings

The savings rate is the most intuitive representation of people’s time preferences. How much a person will save is determined by his or her time preference. The CFPS database does not make survey data on savings and savings rates readily available. Following Ma and Zhou (2014), we calculate the household savings rate as (total household income—total household expenditures)/total household income. Total household expenditures include medical expenditures, food expenditures, education expenditures and entertainment and leisure expenditures. Medical expenditures are closely related to the health statuses of family members and may change suddenly. Education expenditures are affected by whether a family has children. Both of these are rigid expenditure, which means consumption of daily necessities. Therefore, we exclude medical and education expenditures from the total household expenditure when we calculate the household saving rate (excluding medical and education expenditures; *Saving*). To check the stability of the results, we use the total household expenditure, including medical and education expenditures, to calculate the second household saving rate (including medical and education expenditures; *Sav-edu&med*).

#### Measurement of consumption

First, we measure the household consumption rate, excluding medical and education expenditures, as the ratio of total household expenditures (excluding medical and education expenditures) to total household income (*Consumption*). To check the stability of the results, we use the total household expenditure, including medical and education expenditures, to calculate the second household consumption rate (including medical and education expenditures; *Cons-edu&med*). With reference to the empirical study of Ma and Zhou (2014), who report residential consumption expenditures as household expenditures, we use the total residential consumption expenditures as the numerator to calculate the household consumption rate (*Cons-spending*). Second, consumption is classified by purpose into three types: survival consumption (*Cons-survival*), developmental consumption (*Cons-development*) and enjoyment consumption (*Cons-enjoyment*). In this paper, *Cons-survival* is defined as the consumption of food, clothing and housing. *Cons-development* is defined as the consumption of education, transportation and communications. *Cons-enjoyment* is defined as the consumption of entertainment and leisure, household equipment and durable goods and expenditures on other goods and services (Wang et al., 2020).

#### Measurement of education

We use the fuzzy average number of years of schooling (*Education*) and education expenditure as a share of total household expenditure (*Education expenditure*) to measure educational attainment. *Education* represents the average education level in a county, while *Education expenditure* reflects investment in human capital.

#### Measurement of mindset

We analyze people’s perceptions about time preference in terms of their behavior and values. We use the following variables to measure these perceptions: time spent engaging in religious activities on weekdays (*Religious-day*), time spent engaging in religious activities at weekends (*Religious-end*), frequency of physical activity (*Fitness*), belief that intelligence is rewarded (*Intelligence*) and importance given to having fun (*Fun*), passing on the family name (*Offspring*) and having children (*Children*). The first three variables address people’s behavior, while the latter four address their values; their ideologies can be inferred from both behavior and values.

#### Measurement of seismic activity

To facilitate matching with the CFPS data, we use the number of historical earthquakes in each county to measure earthquakes frequency. We use the time of the most recent earthquake to check the stability of the results. We also use the product of the maximum earthquake magnitude and time of occurrence of the maximum magnitude as the interaction term (interaction term: magnitude* time). Furthermore, we also use the product of the number of earthquake occurrences and average magnitude as the interaction term (interaction term: frequency* magnitude). The test metrics are treated logarithmically.

#### Other control variables

Using the control variable selection method introduced by Yao and Xu (2018), we divide the control variables in this study into three categories: regional characteristics, household characteristics and economic characteristics. The CFPS database provides thorough records of household income and expenditures. As we mainly focus on household savings and consumption in this study, the household variables are controlled (Yao et al., 2019a). Drawing on the methods of Ma and Zhou (2014) and Yao et al. (2019a), we select family income (*Family income*), family size (*Family size*), and health status (*Health*) as the main household control variables. We measure *Health* using household medical expenditures.

We also draw on the method of Yao et al. (2019a) to select regional characteristic variables, choosing the total population (*Population*) and the share of non-agricultural population (*Non-agricultural population*) as the main regional control variables.

Economic development affects people’s savings, consumption and human capital investment behavior. We control for economic development at both the provincial and village levels. At the provincial level, economic development is measured using the gross domestic product (*GDP*) and gross domestic product per person (*GDP per person*). At the village level, economic development is measured using the average gross income of the county (*Cincome*)^4^. *GDP* and *GDP per person* are subjected to logarithmic transformation.

## Empirical results

We use the following econometric model to examine the effect of earthquakes on time preferences and thus test the theoretical hypothesis presented in section “Relevant facts and theoretical hypotheses”:


(1) $$Y_{i}=a_{0}+a_{1}\times x_{i}+a_{2}\times c\,o\,n_{i}+\varepsilon_{i}$$

In this formula, the subscript *i* represents the *i*th sample. *Y_i_* represents the household savings rate, household consumption rate, personal ideology and education level of the *i*th sample. *a*_0_ is a constant term. *x*_*i*_ represents the number of earthquakes. *con_i_* represents the household, regional and economic characteristic control variables. ε*_i_* is the random disturbance term. Ordinary least squares (OLS) regression is used in this model.

### Evidentiary results and explanations

Using the data and sample defined above, we test the effect of historical earthquakes on people’s time preferences by regressing several variables that capture changes in people’s mindset of time preferences: household savings rate, household consumption rate and average years of schooling.

#### Baseline regression results

Table 2 reports the results of baseline regressions of the effects of historical earthquakes on several variables representing time preferences. Columns (1) to (3) present the results obtained using a simple model, and columns (4) to (6) present the results obtained when the control variables are included. The results show that the effects of the number of earthquakes on the household saving rate, household consumption rate and mean years of schooling are significant at the 1% level, regardless of whether the control variables are included in the model.

**TABLE 2:** Effect of earthquakes on time preferences.

	Saving	Consumption	Education	Saving	Consumption	Education
						
	(1)	(2)	(3)	(4)	(5)	(6)
Number of earthquakes	−0.018***	0.018***	−0.182***	−0.017***	0.017***	−0.138***
	(0.006)	(0.006)	(0.008)	(0.006)	(0.006)	(0.008)
Control variables	NO	NO	NO	YES	YES	YES
Sample size	2164	2164	2456	2164	2164	2316
*R*-squared	0.004	0.004	0.165	0.065	0.065	0.588

***p < 0.01.

Specifically, columns (1) and (4) estimate the impact of historical earthquakes on *Saving*. The addition of the control variable in column (4) results in an estimated coefficient of –0.017, which is significant at the 1% level. This result indicates that areas with more historical earthquakes have a lower household saving rate than areas with fewer earthquakes (i.e., the number of earthquakes is negatively correlated with the household saving rate). Columns (2) and (5) examine the impact of historical earthquakes on *Consumption*. The addition of the control variable in column (5) results in an estimated coefficient that is positive and significant at the 1% level, indicating that on average, household consumption in the region increases by 1.7% for each one-unit increase in the number of earthquakes. In other words, the number of historical earthquakes is positively correlated with the household consumption rate. Finally, columns (3) and (6) examine the impact of historical earthquakes on *Education*. The addition of control variables in column (6) results in an estimated coefficient of –0.138, which is significant at the 1% level. This result indicates that the number of historical earthquakes is negatively correlated with the number of years of education and thus with human capital investment, such that the average number of years of education in the region decreased by 0.138 years for each one-unit increase in the number of earthquakes.

The results show that in areas exposed to more historical earthquakes, the household consumption rate tends to increase while the household savings rate and average years of education tend to decrease. These findings are consistent with our hypothesis that uncertainty due to historical earthquakes affects people’s time preferences and induces changes in their savings, consumption and education behavior, reflecting an ideological mindset of “living for today.” However, the mechanism through which historical earthquakes exert this effect remains to be analyzed and verified.

#### Discussion on consumption classification

We classify consumption as survival, enjoyment or development consumption according to the definitions in section “Measurement of consumption” and Wang et al. (2020). We expect to find that households in areas frequently exposed to earthquakes increase their survival and enjoyment consumption and decrease their development consumption. This pattern would reflect a tendency to focus on the present and personal enjoyment rather than on self-development as a consequence of the “living for today” mindset.

Table 3 presents the empirical results, which are in line with our expectations. Specifically, when control variables are included in the model, the estimated coefficients of survival and enjoyment consumption are 0.016 and 0.009, respectively, and these are positive and significant at the 1 and 10% levels, respectively. These results indicate that in regions with more historical earthquakes, each additional earthquake is associated with a 1.6 and 0.9% increase in household survival and enjoyment consumption, respectively. The coefficient of development consumption is −0.009, which is significant at the 1% level. This result indicates that in regions with more historical earthquakes, the household development consumption rate decreases by 0.9% for each additional earthquake. This also shows that economic factors alone cannot explain significantly negative estimated coefficient of developmental consumption. This is because if earthquakes affect people’s time preference mainly by affecting the economic level, then in the discussion of consumption classification, survival, enjoyment and developmental consumption should show a homogeneous trend, that is, increase or decrease together. However, the results show that earthquake frequency positively affects survival consumption and enjoyment consumption, while negatively affects developmental consumption. This also suggests, to some extent, that there are other factors influencing people’s time preferences. One possible explanation is that the earthquakes have influenced people’s mindset and influenced their consumption and behavior by the concept of “living in the moment.”

**TABLE 3:** Earthquakes and different types of consumption.

	Cons-survival		Cons-enjoyment		Cons-development	
			
	(1)	(2)	(3)	(4)	(5)	(6)
Number of earthquakes	0.017***	0.016***	0.012**	0.009*	−0.007***	−0.009***
	(0.003)	(0.003)	(0.005)	(0.003)	(0.002)	(0.002)
Control variables	NO	YES	NO	YES	NO	YES
Sample size	2262	2254	2331	2316	2289	2277
*R*-squared	0.014	0.103	0.003	0.009	0.006	0.041

*p < 0.1, **p < 0.05, ***p < 0.01.

These results of our regression of earthquakes frequency on the household saving rate, household consumption rate, average years of education and types of consumption are consistent with our conjecture that people who live in areas that have long been affected by earthquakes cherish and want to enjoy their current lives. Accordingly, they consume more and save less and pay less attention to education and self-development than people in less earthquake-prone areas do.

### Robustness tests

In this paper, we apply three tests of robustness. First, we construct new indicators that capture the explanatory variables (savings, consumption, and education). Second, we construct two new earthquake indicators, using the time of the most recent earthquake, the interaction term between the maximum earthquake magnitude and the time of this magnitude to measure the robustness of the earthquake and the interaction term between the number of earthquake occurrences and average magnitude (both variables are logarithmically treated). We expect both variables to affect people’s time preferences (i.e., patience reduction). A plausible interpretation of the first indicator is that the more recent the occurrence of an earthquake, the more that people in the area remember the related threat of death. People affected by such negative emotions have reduced self-control (Zhang and Liu, 2021), and they prefer to enjoy life, increase their consumption and reduce their savings and human capital investment. Additionally, the largest earthquake that has ever occurred in a region poses a profound threat of death. Although the largest earthquakes did not occur recently, the impact of such uncertainty can have subtle long-term effects on the temporal preferences of people in the region. As for the third indicator, since both the frequency and magnitude of earthquakes have corresponding effects on people in a region (Zhang and Liu, 2021; Yu, 2022), we use the interaction term of the two as a robustness check indicator. Finally, we reduce the period in which we analyze the occurrence of earthquakes. We apply robustness tests to the baseline regression results from samples of earthquakes that have occurred since 1900 and since 1368 (i.e., the establishment of the Ming dynasty).

#### Savings

Our previously described analyses confirm that the number of historical earthquakes negatively affects the savings rate of households in the region. Table 4 shows the results of robustness tests. In column (1), we replace *Saving* with *Sav-edu&med*. In columns (2–4), we replace the original earthquake indicator with two robustness indexes: the time of the most recent earthquake and the interaction term between the maximum earthquake magnitude and the time of this magnitude, respectively. Consistent with our previous analyses, we find that the impact of earthquake uncertainty on household savings rates is negative and significant. Specifically, the most recent earthquake, the largest earthquake in history and magnitude negatively affect people’s saving behavior.

**TABLE 4:** Robustness test: earthquakes and savings.

	Sav-edu&med	Saving	Saving	Saving
				
	(1)	(2)	(3)	(4)
Number of earthquakes	−0.015* (0.008)			
Recent earthquake time		−0.106** (0.046)		
Interaction item: magnitude* time			−0.126*** (0.039)	
Interaction item: magnitude* frequency				−0.056** (0.023)
Control variables	YES	YES	YES	YES
Sample size	2164	2164	2107	2164
*R*-squared	0.161	0.064	0.066	0.064

*p < 0.1, **p < 0.05, ***p < 0.01.

#### Consumption

As in the previous section, we replace the explained variable (*Saving, Consumption, and Education*) and explanatory variable (*Earthquakes frequency*) separately using the replacement variable method. In addition to *Cons-edu&med*, we construct a household consumption rate by summing consumer spending. Consistent with our conjecture, earthquake uncertainty has a positive impact on household consumption rate in Table 5. Specifically, the most recent earthquake, the largest earthquake in history and magnitude positively affect people’s consumption behavior.

**TABLE 5:** Robustness test: earthquakes and consumption.

	Cons-edu&med	Cons-spending	Consumption	Consumption	Consumption
					
	(1)	(2)	(3)	(4)	(5)
Number of earthquakes	0.015* (0.008)	0.020*** (0.006)			
Recent earthquake time			0.106** (0.046)		
Interaction item: magnitude* time				0.126*** (0.038)	
Interaction item: magnitude* frequency					0.056*** (0.023)
Control variables	YES	YES	YES	YES	YES
Sample size	2164	2188	2164	2107	2164
*R*-squared	0.161	0.195	0.064	0.066	0.064

*p < 0.1, **p < 0.05, ***p < 0.01.

#### Education

As in the first two robustness tests, we replace *Education* with *Education expenditure*. In columns (2–4), we replace the original earthquake index with three robustness indexes in: the time of the most recent earthquake and the interaction term between the maximum earthquake magnitude and the time when this magnitude occurred, respectively. Table 6 shows that historical earthquakes have a negative impact on human capital investment. Again, this result is consistent with our conjecture that the most recent earthquake, the largest earthquake in history and magnitude negatively impact people’s human capital investment behavior.

**TABLE 6:** Robustness test: earthquakes and education.

	Education expenditure	Education	Education	Education
				
	(1)	(2)	(3)	(4)
Number of earthquakes	−0.006*** (0.046)			
Recent earthquake time		−0.906*** (0.046)		
Interaction item: magnitude* time			−0.822*** (0.039)	
Interaction item: magnitude* frequency				0.056*** (0.023)
Control variables	YES	YES	YES	YES
Sample size	2300	2316	2258	2316
*R*-squared	0.025	0.572	0.586	0.576

*p < 0.1, ***p < 0.01.

#### Sample of earthquakes at different times

Our original sample of seismic data from 780 BC to 1979 AD is large. To test the robustness of our results, we screen two samples with shorter time periods: (1) earthquakes occurring since 1368 (i.e., the establishment of the Ming Dynasty), and (2) earthquakes occurring since 1900. As shown in Table 7, our results remain robust, indicating that exposure to historical earthquakes influences people’s time preferences by reducing their patience.

**TABLE 7:** Robustness teat: earthquake samples over periods of different lengths.

	After 1368			After 1900		
	Saving	Consumption	Education	Saving	Consumption	Education
						
	(1)	(2)	(3)	(4)	(5)	(6)
Number of earthquakes	−0.015**	0.015**	−0.108***	−0.025***	0.025***	−0.136***
	(0.007)	(0.007)	(0.007)	(0.009)	(0.009)	(0.010)
Control variables	YES	YES	YES	YES	YES	YES
Sample size	1851	1851	1982	1115	1115	1188
*R*-squared	0.061	0.061	0.628	0.059	0.059	0.549

**p < 0.05, ***p < 0.01.

## Further analysis

In this section, we further analyze the heterogeneous effects of variations in earthquake magnitudes and frequency and the effects of earthquakes on ideological perceptions.

### Heterogeneous effects of earthquake levels

The number of earthquakes in a set period is a measure of earthquake frequency. However, some regions may have a high frequency of low-magnitude earthquakes, whereas other regions may have a low frequency of high-magnitude earthquakes. Generally, the higher the magnitude of the earthquake, the greater the threat of death. Small and frequent earthquakes may encourage people to “prepare for a rainy day.” Therefore, our selection of the number of earthquakes over time as our measure of historical earthquakes may not be optimal. Therefore, we categorize both the frequency of earthquakes of magnitude 4.5^5^ or greater and the average magnitude of earthquakes by percentage (50%) to examine the effects of both frequency and magnitude on people’s temporal preferences.

Table 8 examines the effects of the frequency of earthquakes on people’s saving, consumption and human capital investment behavior. Part A of the table presents the results of analysis of the frequency of earthquakes of magnitude 4.5 or above in the top 50%, i.e., the impact of a low frequency of earthquakes on people’s time preferences. Part B presents the results of analysis of the frequency of earthquakes of magnitude 4.5 and above in the bottom 50%, i.e., the impact of a high earthquake frequency on people’s time preferences. The results show that whereas a low earthquake frequency may not affect people’s saving and consumption behavior, a high earthquake frequency significantly affects people’s saving, consumption and human capital investment behavior. Specifically, in Part B, the estimated coefficients of *Savings* and *Education* are significantly negative at the 10 and 1% levels, respectively, while the estimated coefficient of *Consumption* is significantly positive at the 1% level. It is worth noting that the effect of low earthquake frequency on years of education is significantly positive in column (3). One possible explanation is that low earthquake frequency does not bring a strong threat of death to people and brings a risk aversion. People living in such places will try to counteract the risk posed by earthquakes through education.

**TABLE 8:** Heterogeneity test: Number of earthquake occurrences.

	A: Frequency of 4.5 magnitude earthquakes (top 50%)			B: Frequency of 4.5 magnitude earthquakes (back 50%)		
	Saving	Consumption	Education	Saving	Consumption	Education
						
	(1)	(2)	(3)	(4)	(5)	(6)
Frequency of 4.5 magnitude earthquakes	−0.023	0.023	0.317***	−0.033*	0.033*	−0.145***
	(0.044)	(0.044)	(0.021)	(0.017)	(0.017)	(0.018)
Control variables	YES	YES	YES	YES	YES	YES
Sample size	1182	1182	1276	982	982	1040
*R*-squared	0.084	0.084	0.651	0.045	0.045	0.697

*p < 0.1, ***p < 0.01.

Table 9 presents the results of analyses of the effects of earthquake magnitude on people’s saving, consumption and human capital investment behavior. In part A, the results are presented for the top 50% of average magnitudes; that is, we examine the effect of small earthquakes on people’s time preferences. In part B, the results are presented for the bottom 50% of average magnitudes; that is, we examine the effect of large earthquakes on people’s time preferences. Again, the results show that while small earthquakes may not affect people’s saving and consumption behavior, large earthquakes significantly affect people’s saving, consumption and human capital investment behavior. Specifically, in Part B, the estimated coefficients of *Saving* and *Education* are negative and significant at the 5 and 1% levels, respectively, and the estimated coefficient of *Consumption* is significantly positive at the 1% level. It is also worth noting that small and large earthquakes have opposite effects on household saving and consumption rates. This is because when the probability of earthquake-induced death is small, the “property risk effect” is dominant and people tend to save more and consume less; when the probability of earthquake-induced death is large, the “life risk effect” is dominant and people tend to save more and consume less (Yu et al.). When the probability of earthquake-induced death is high, the “life risk effect” is dominant and people tend to increase consumption and decrease savings (Yu et al.)

**TABLE 9:** Heterogeneity test: mean magnitude.

	A: mean magnitude (top 50%)			B: mean magnitude (back 50%)		
	Saving	Consumption	Education	Saving	Consumption	Education
						
	(1)	(2)	(3)	(4)	(5)	(6)
Average magnitude of earthquake	0.004***	−0.004***	−0.014***	−0.005**	0.005**	−0.054***
	(0.002)	(0.002)	(0.005)	(0.002)	(0.002)	(0.001)
Control variables	YES	YES	YES	YES	YES	YES
Sample size	1074	1074	1205	1090	1090	1111
*R*-squared	0.067	0.067	0.487	0.114	0.114	0.679

**p < 0.05, ***p < 0.01.

The results in Tables 8, 9 indicate that although low-magnitude earthquakes may not significantly affect people’s time preferences, quantitative changes lead to qualitative changes such that frequent small earthquakes may still significantly affect people’s savings, consumption and educational behavior. Zhang and Liu (2021) verify that an increase in the frequency of exposure to earthquakes is significantly associated with a person’s negative emotions. Although small earthquakes are unlikely to cause serious bodily harm to residents, repeated earthquakes and the associated threat of death increase people’s negative emotions, such as fear and anxiety, which may increase their desire to live in the moment. This result also partly explains the rationale for using earthquake frequency to measure historical exposure to earthquakes. Furthermore, in Part B, the estimated coefficients in Table 8 are statistically significantly higher than those in Table 9 by about 3–5 times, suggesting that earthquake frequency has a stronger impact on people’s time preferences than magnitude. Therefore, we use the number of earthquakes as the main measure of historical exposure to earthquakes.

### Earthquakes, mindset and economy

#### Earthquakes and mindset

In addition to incurring huge economic losses, earthquakes have major psychological effects on victims (Kılıç and Ulusoy, 2003) and pose a risk of death (Zhang and Liu, 2021; Yu, 2022). Over time, earthquakes have profound, intergenerational effects on the humanistic environment and mindset of a region (Yu, 2022). If earthquakes affect people’s time preferences, then they should also affect ideologies related to time concepts. People who live in earthquake-prone areas for long periods tend to report a higher perceived mortality risk (Zhang and Liu, 2021) and lower patience levels. The threat of death due to disaster shocks is believed to have a psychological impact on victims and induce changes in their time preferences (Greenberg et al., 1986; Arndt et al., 2004; Sawada and Kuroishi, 2015; Cassar et al., 2017; Filipski et al., 2019).

Many scholars confirm that the psychological effect of the risk of death reduces people’s patience, leading them to save less and spend more. For example, Levhari and Mirman (1977) confirms that people exposed to an elevated risk of death prefer to increase their current (vs. future) consumption. Yaari (1965) and Filipski et al. (2019) observe that people’s patience level decreases in response to the risk of death. Historical earthquakes represent a shock to people’s mindsets and increase the risk of death, possibly leading to reductions in savings and human capital investment and an increase in consumption. Such changes would reflect a shift in time preferences, namely a decrease in patience.

Accordingly, we expect historical earthquakes to affect people’s conceptualization of time. In this study, we select and test items from the CFPS (2010) questionnaire that reflect people’s values related to time preference. Table 10 presents the results of our OLS benchmark regression of Equation 1, which estimates the impact of historical earthquakes on ideology. First, we use the analyses in columns (1–3) to validate people’s behavior. In columns (1, 2), the estimated coefficients of *Religious-day* and *Religious-end* are 0.1 and 0.062 and are significant at the 1 and 5% levels, respectively. The results indicate that with each one-unit increase in the number of earthquakes, *Religious-day* and *Religious-end* increase by 10.3 and 6%, respectively. That is, people’s enthusiasm for religious activities increases. The estimated coefficient of column (3) is –0.021 and is significant at the 1% level, which indicates that with each increase in the number of earthquakes, *Fitness* decreases by 2.1%; in other words, increased exposure to historical earthquakes reduces the attention paid to maintaining one’s physical health and fitness. In columns (4–7), we verify the effects of historical earthquakes on people’s value orientation. The first variable examines people’s perceptions of personal ability. We find that the number of historical earthquakes is negatively associated with *Intelligence*. The second variable examines people’s perceptions of pleasure in life. The results show that the frequency of earthquakes is positively associated with *Fun*, suggesting an increased desire to enjoy life. The third and fourth variables test people’s views on future generations. Similarly, an increase in historical earthquakes is negatively associated with *Offspring* and *Children*, suggesting a decrease in people’s attention to future generations.

**TABLE 10:** Earthquakes and mindsets.

	Religious-day	Religious-end	Fitness	Intelligence	Fun	Offspring	Children
							
	(1)	(2)	(3)	(4)	(5)	(6)	(7)
Number of earthquakes	0.100***	0.062**	−0.021*	−0.017***	0.010**	−0.027***	−0.010***
	(0.034)	(0.031)	(0.011)	(0.004)	(0.004)	(0.005)	(0.004)
Control variables	YES	YES	YES	YES	YES	YES	YES
Sample size	120	131	755	6177	6177	6177	6177
*R*-squared	0.192	0.151	0.022	0.010	0.027	0.019	0.014

*p < 0.1, **p < 0.05, ***p < 0.01.

The results in this section show as the frequency of earthquakes increases, people increase their engagement in religious activities while paying less attention to their fitness, their personal abilities and future generations. The results are consistent with our conjecture that an increasing frequency of earthquakes is associated with an increasing emphasis on “living for today” mindset. As a result, people focus on the present and aim to enjoy life rather than improve themselves.

#### Earthquakes and economy

In addition to differences in mindset that may be an important reason for the above-mentioned differences in people’s time preferences and behaviors, the level of economic development may also have an impact on people’s time preferences (Zhang et al., 2017). One possible explanation is that the frequency of earthquakes leads to a region’s poor level of economic development, which further affects people’s saving, consumption, and education behaviors. Although we have controlled for provincial income and village-level income, which represents the economic level in the baseline regression, we also tested the relationship between earthquakes, economic level, and time preference to further analyze the mechanism of the effect of earthquakes on time preference.

The results in Table 11 support reviewer’s hypothesis that the frequency of earthquakes is negatively related to the level of economic development. The more frequent earthquakes occur, the lower the level of economic development of the region. In turn, the level of economic development is positively related to the household savings rate and the average years of schooling, and negatively related to the household consumption rate. This suggests that historical earthquakes do affect people’s saving, consumption and education behavior by affecting the level of economic development in a region. Areas that have been hit by earthquakes for a long time are limited by geographic resources and environment and are economically underdeveloped. People in economically disadvantaged areas prefer to spend money rather than save or develop themselves.

**TABLE 11:** Earthquake, economy and time preference.

	Economic level	Saving	Consumption	Education
				
	(1)	(2)	(3)	(4)
Number of earthquakes	−0.487*** (0.084)			
Economic level		0.001** (0.001)	−0.001** (0.001)	0.012*** (0.001)
Control variables	YES	YES	YES	YES
Sample size	2316	2300	2316	2258
*R*-squared	0.572	0.025	0.572	0.586

**p < 0.05, ***p < 0.01.

Whether it is the mindset or the level of economic development that has a greater impact on people’s time preference, however, is not determined in our current study. However, given the different effects of earthquakes on different types of consumption, we prefer that ideology has a greater impact on people’s time preference.

## Conclusion and implications

China is prone to frequent earthquakes because of its location between two major seismic zones, and the areas affected by earthquakes are large. Uncertainty due to frequent earthquakes can permanently shape people’s time preferences and behavior. Therefore, this paper explores the impact of historical earthquakes on people’s time preferences from a historical perspective. Using data from the 2010 CFPS database and the China Historical Earthquake Catalog, this paper studies the impact of historical earthquakes on savings rates, consumption rates and educational behavior. We find that, on average, the household savings rates and regional average years of education decreased with each additional earthquake, indicating that historical earthquakes have a negative impact on people’s saving and human capital investment behavior. In contrast, the household consumption rate increases with each additional earthquake, which means that historical earthquakes positively affect people’s consumption behavior. We further find that exposure to earthquakes promotes survival and enjoyment consumption and inhibits development consumption. To further support our hypothesis, we verify that frequent historical earthquakes affect people’s conceptualization of time and promote religious beliefs, while reducing their sense of achievement and concern about future generations; in other words, our findings indicate a present-focused mindset. We also prove that the uncertainty caused by a high frequency of earthquakes has a greater impact on people’s time preferences than exposure to high-magnitude earthquakes.

The observed phenomena are all related to the “living for today” ideology. Over time, the disruption caused by earthquakes may lead people to contemplate separation from loved ones in life or death. The prospect of death may elicit thoughts such as “You never know—tomorrow or an accident, which will come first?” Such thoughts lead people to cherish their present lives. Such psychological factors can affect time preferences, leading people to increase their enjoyment of life and present consumption and reduce their focus on the future. Additionally, these factors cause them to pay less attention to education and saving.

We are inspired to present this research because although China is deeply affected by earthquakes, the government and society are more concerned about economic and post-disaster reconstruction efforts after a single earthquake, and less concerned about the long-term impact of the uncertainty caused by frequent earthquakes on a region. This uncertainty is somewhat similar to the current uncertainty caused by repeated outbreaks of the ongoing COVID-19 pandemic. Our conclusions herein have implications in terms of predicting the impact of the COVID-19 pandemic on people’s time preferences. We believe that governments and societies should pay more attention to the long-term impact of uncertainty caused by frequent natural disasters on a region and study the human impact of such natural disasters from a psychological perspective.

## Data availability statement

The datasets presented in this study can be found in online repositories. The names of the repository/repositories and accession number(s) can be found below: 1. https://data.earthquake.cn/datashare/report.shtml?PAGEID=datasourcelist&dt=8a85efd754e7d6910154e7d691810000. 2. http://www.isss.pku.edu.cn/cfps/.

## Author contributions

CD contributed to the conception of the study and helped perform the analysis with constructive discussions. XY performed the experiment, contributed significantly to analysis and manuscript preparation, performed the data analyses, and wrote the manuscript. ZC helped perform the analysis with constructive discussions and helped in data analysis. All authors contributed to the article and approved the submitted version.

## Footnotes

1 Zhang and Liu (2021) divide China into regions with earthquakes and those without earthquakes, because their dataset starts in 1900 AD and the regions in which earthquakes have occurred are easily distinguished from those in which no earthquakes have occurred. In contrast, our data start in the BC era, and during our sample period only one region has never experienced an earthquake. Therefore, we modify the division criteria slightly to make it easier to distinguish the regions. According to data from the Chinese historical earthquake catalog, seven regions (Gansu, Qinghai, Hebei, Sichuan, Yunnan, Xinjiang, and Tibet) have had at least 100 earthquakes over time. We classify these regions as earthquake-prone and the rest as non-earthquake-prone.

2 Quoted from the speech given by Mr. Ai, Vice Chairman of the Standing Committee of the National People’s Congress (NPC), at the sixth meeting of the Standing Committee of the 13th NPC.

3 A few points need to be clarified. First, in the analysis of ideology, we use data on adults from the CFPS, whereas the other analyses use data on families in the CFPS. Additionally, the size of the sample used to study ideology is different from that of the samples used in the other analyses. Second, because the data on total household income, medical expenditure and fuzzy total population are large values, we divide these three indicators by 1000, 1,000, and 100,000, respectively, for the convenience of regression.

4 We classify each village based on its identifier (CID), aggregate the total household income of the village and obtain the average income of each village.

5 According to earthquake classification, earthquakes with a magnitude greater than 3 and less than 4.5 are called felt earthquakes and usually do not cause damage. Earthquakes with a magnitude greater than 4.5 and less than 6 are called moderate-to-strong earthquakes and are classified as destructive earthquakes. Because China’s historical earthquake catalog counts information about earthquakes of magnitude 4 and above, considering that earthquakes with a magnitude of 4.5 may be more destructive and may have a greater impact on people’s time preferences, we selected a magnitude of 4.5. Earthquake frequency replaces the number of earthquakes.
